# Esthesioneuroblastoma

**DOI:** 10.1590/0100-3984.2015.0206

**Published:** 2017

**Authors:** Aline de Araújo Naves, Luiz Gonzaga da Silveira Filho, Renata Etchebehere, Hélio Antônio Ribeiro Júnior, Francisco Valtenor A. Lima Junior

**Affiliations:** 1 Universidade Federal do Triângulo Mineiro (UFTM), Uberaba, MG, Brazil.; 2 Hospital das Clínicas da Faculdade de Medicina de Ribeirão Preto da Universidade de São Paulo (HCFMRP-USP), Ribeirão Preto, SP, Brazil.

**Figure 1:**
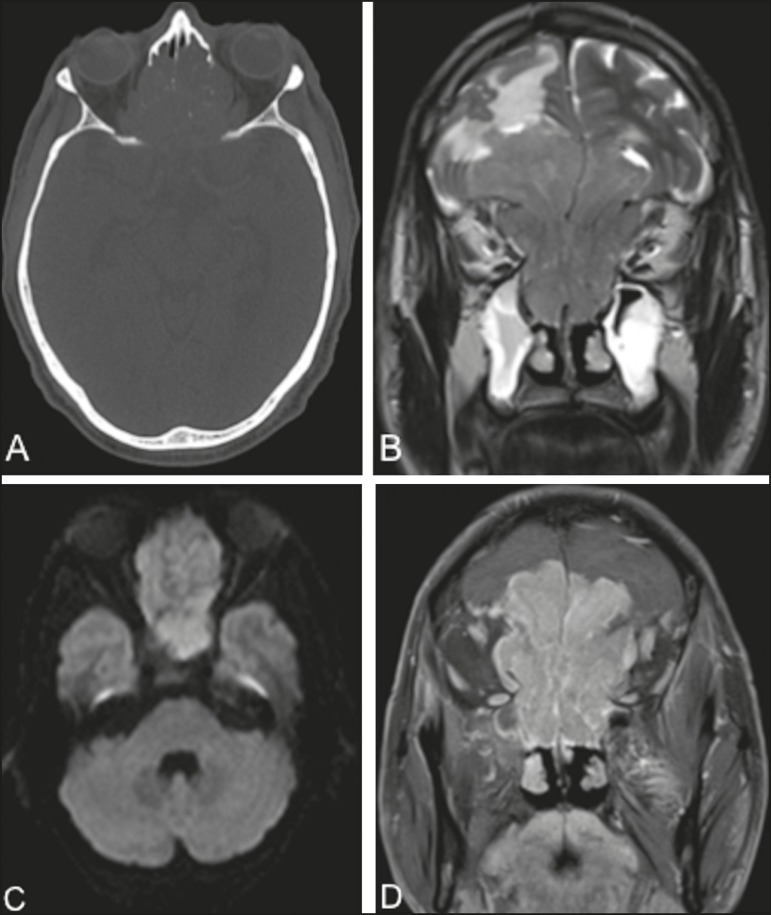
CT of the brain (A), with a bone window, showing an expansile lesion occupying ethmoid cells and containing calcifications, with bone destruction. MRI demonstrated that the lesion was extra-axial, with lobulated contours, located in the upper portion of the nasal cavity, and extended to the anterior cranial fossa, facial sinuses, and orbits. A coronal T2-weighted sequence (B) shows that the expansile lesion presented an isointense signal, although a hyperintense signal (edema) can be seen in the brain parenchyma in the frontal lobe, mainly on the left. An axial diffusion-weighted imaging sequence (C) shows a hyperintense signal (restricted diffusion). A contrast-enhanced coronal T1-weighted sequence (D) shows intense enhancement.

Dear Editor,

A 64-year-old male presented with nasal obstruction, anosmia, and a reduction in visual acuity over the last few months, together with weight loss and a two-year history of headache. Computed tomography (CT) of the brain (Figure 1A) showed an expansile lesion with poorly defined borders, occupying the ethmoid cells, sphenoid sinuses, and the anterior cranial fossa, accompanied by edema of the frontal lobes. On magnetic resonance imaging (MRI) scans (Figures 1B, 1C, and 1D), the lesion showed restricted diffusion and intense enhancement after contrast administration. A biopsy was performed, and analysis of the biopsy sample revealed hyperchromatic cells organized around a fibrillar stroma, forming rosettes, consistent with a diagnosis of olfactory neuroblastoma. The lesion was staged histologically as grade I in the Hyams grading system. There was no evidence of cervical involvement or distant metastases. The patient died 15 days after undergoing the examinations.

Olfactory neuroblastoma, also known as esthesioneuroblatoma, is a rare malignant neoplasm of neuroectodermal origin and accounts for 3–6% of all malignant tumors of the paranasal sinuses. It has a bimodal age distribution, being most common among adults in the second or fifth decades of life^(1)^. It is believed that the neoplasm arises from the olfactory epithelium, originating in the superior portion of the nasal cavities, ascending across the cribriform plate, and extending into the anterior cranial fossa^(2)^.

Clinically, olfactory neuroblastoma manifests as nasal obstruction or epistaxis. It can show indolent behavior, promote local invasion, and generate distant metastases. It tends to invade the paranasal sinuses, orbits, and anterior cranial fossa. The most common metastases are to the lymph nodes of the neck, lungs, liver, and bone, such dissemination at the time of diagnosis being the main predictor of survival^(2)^. Although there is no universally accepted staging system, the Kadish classification system, established in 1976 and considered an important prognostic predictor, is widely used. In the Kadish system, stage A indicates that the tumor is limited to the nasal cavity; stage B indicates that it involves only the nasal cavity and paranasal sinuses; and stage C indicates that it extends beyond the stage B limits. The staging system proposed by Dulguerov employs the tumor-node-metastasis classification^(3,4)^.

Bone destruction and calcification within the lesion can be characterized by CT^(5)^. An MRI scan provides more accurate information on the extent of the tumor, especially in terms of intracranial and orbital involvement. On MRI, the majority of olfactory neuroblastomas present a signal that is (in relation to that of muscle tissue) hypointense in T1-weighted sequences and hyperintense in T2-weighted sequences, as well as showing intense enhancement in contrast-enhanced sequences^(6,7)^. MRI is also superior to CT in the evaluation of recurrence after craniofacial resection, because of its greater ability to differentiate fibrous scar tissue from residual or recurring neoplasia^(6)^. Cysts in the intracranial margin of the tumor have been reported in cases of olfactory neuroblastoma. Another relevant aspect is a dumbbell-like morphology, the tumor mass being divided between the anterior cranial fossa and the nasal cavity, the cribriform plate forming the “waist”^(5)^.

The main differential diagnoses of olfactory neuroblastoma include: squamous cell carcinoma, typically in the maxillary antrum, with bone erosion; sinonasal adenocarcinoma, with heterogeneous enhancement, which has been associated with occupational exposure to wood dust; undifferentiated sinonasal carcinoma, which affects older patients; and dural-based invasive meningioma, with poorly defined borders and areas of necrosis^(8)^.
